# Immunosenescence and Inflammation in Chronic Obstructive Pulmonary Disease: A Systematic Review

**DOI:** 10.3390/jcm13123449

**Published:** 2024-06-13

**Authors:** Fabíola Ramos Jesus, Fabine Correia Passos, Michelle Miranda Lopes Falcão, Marcelo Vincenzo Sarno Filho, Ingrid Lorena Neves da Silva, Anna Clara Santiago Moraes, Margarida Célia Lima Costa Neves, Gyselle Chrystina Baccan

**Affiliations:** 1 Maternidade Climério de Oliveira (MCO/EBSERH), Universidade Federal da Bahia, Salvador 40055-150, Bahia, Brazil; fabiola.jesus@ebserh.gov.br; 2 Departamento de Bioquímica e Biofísica, Instituto de Ciências da Saúde, Universidade Federal da Bahia, Salvador 40110-110, Bahia, Brazil; 3 Departamento de Saúde, Universidade Estadual de Feira de Santana, Avenida Transnordestina, s/n—Novo Horizonte, Feira de Santana 44036-900, Bahia, Brazil; 4 Unidade do Sistema Respiratório, Ambulatório Professor Francisco Magalhães Neto-Hospital Universitário Professor Edgard Santos, Universidade Federal da Bahia, Salvador 40110-200, Bahia, Brazil

**Keywords:** immunosenescence, aging, COPD, systematic review

## Abstract

**Background/Objectives**: Chronic Obstructive Pulmonary Disease (COPD) is a disease of premature aging, characterized by airflow limitations in the lungs and systemic chronic inflammation. This systematic review aimed to provide a systematic overview of immunosenescence and inflammation in Chronic Obstructive Pulmonary Disease (COPD). **Methods:** The PubMed, Science Direct, Scopus, Cochrane Library, and Web of Science databases were searched for studies on markers of immunosenescence. Observational studies comparing patients with COPD to individuals without disease were evaluated, considering the following markers: inflammation and senescence in COPD, naïve, memory, and CD28^null^ T cells, and telomere length in leukocytes. **Results:** A total of 15 studies were included, eight of which were rated as high quality. IL-6 production, telomere shortening, and the higher frequencies of CD28^null^ T cells were more prominent findings in the COPD studies analyzed. Despite lung function severity being commonly investigated in the included studies, the importance of this clinical marker to immunosenescence remains inconclusive. **Conclusions:** The findings of this systematic review confirmed the presence of accelerated immunosenescence, in addition to systemic inflammation, in stable COPD patients. Further studies are necessary to more comprehensively evaluate the impact of immunosenescence on lung function in COPD.

## 1. Introduction

COPD, which is mainly caused by the inhalation of noxious particles or gases, leads to airflow limitations that are not fully reversible due to the presence of small airway diseases and emphysema [1,2]. Smoking, one of the main causal agents for COPD development, when associated with a genetic predisposition, may contribute to the risk of disease development [3]. Thought to be associated with premature aging [4], the prevalence of COPD increases with advancing age [5].

Physiological changes in the immune system can occur during senescence, including reductions in the populations of naïve T cells, potentially leading to increased susceptibility to infection [6], higher concentrations of memory T cells, and the development of a chronic low-grade inflammation known as “inflammaging” [7]. Metabolism associated with epigenetic pathways plays a crucial role in immunosenescence. However, the molecular mechanisms that lead to the aging of the immune system are not yet completely understood [8]. In addition to the association between inflammation and age-related morbidity and mortality, chronic inflammatory diseases may accelerate aging, pointing to interactions between immunosenescence and age-related pathologies [9]. The presence of chronic inflammation in senescence has been attributed to factors such as lifestyle, smoking, visceral obesity, intestinal dysbiosis, diet, and physical inactivity [10]. Smoking accelerates the aging of various organs [11]. However, regarding the immune system, moderate tobacco consumption in individuals without lung disease can lead to physiological immunosenescence [12].

Aging markers, including genomic instability, telomere shortening, epigenetic changes, nutritional alterations, mitochondrial dysfunction, and immune dysregulation have been described in COPD [13]. In COPD, as well as in the context of aging, increased pro-inflammatory cytokine production, in addition to other immune changes that lead to the stimulation of innate immune response, results in the development of a pro-inflammatory state [14]. As inflammation has been associated with both COPD and aging, some authors argue that patients may develop “accelerated aging” [14]. Moreover, “inflammaging” has been reported to play a critical role in age-related lung damage and the development of COPD [15,16]. In addition, telomere shortening has been associated with the worsening of gas exchange, pulmonary hyperinflation, and increased risk of mortality [17]. In the context of aging, this research systematically reviews previous studies that evaluated immunosenescence and inflammation in COPD and the implications of the disease.

## 2. Materials and Methods

### 2.1. Protocol

The present systematic review (SR) was conducted in accordance with the guidelines established by the Preferred Reporting Items for SRs and Meta-Analyses PRISMA statement [18] and registered on the PROSPERO online database (CRD42022353600).

### 2.2. Search Strategy

A systematic search of the literature was performed using the PubMed, Cochrane, Scopus, Web of Science, and Science Direct databases from their respective dates of inception until 15 October 2022. The search strategy included only MeSH and DeCS terms. No publication date restrictions were observed, publication language was limited to English, and the grey literature was also searched. For this SR, the following biomarkers were investigated: leukocyte telomere length (TL), systemic inflammation characterized by the presence of pro-inflammatory cytokines IL-6 and IL-8, and absolute or relative frequencies of memory and naïve T cells, as well as markers of T cell senescence (CD28^null^ = CD28^-^).

All initial records from searching the five electronic databases were imported into the Rayyan web-based systematic review software program [19]. After removing duplicates, title and abstract screening was performed independently by two reviewers to exclude non-relevant articles. The articles with titles and abstracts consistent with the eligibility criteria that achieved consensus between the two reviewers were read in full for subsequent inclusion or exclusion in the SR.

Immunosenescence markers and inflammation were categorized into ‘marker types’ according to the definition of immune system senescence found in the literature. A description search strategy was designed using Boolean operators (AND, OR) to determine relevant studies about immunosenescence, inflammation, and COPD. Searches were conducted for all possible combinations of terms: (senescence) AND (IL-6) AND ((COPD) OR (emphysema) OR (chronic bronchitis)); (senescence) AND (IL-8) AND ((COPD) OR (emphysema) OR (chronic bronchitis)); (senescence) AND (telomere) AND ((COPD) OR (emphysema) OR (chronic bronchitis)); (senescence) AND (t cells) AND ((COPD) OR (emphysema) OR (chronic bronchitis)). A standardized checklist was used to ensure that the included texts met the inclusion criteria.

### 2.3. Eligibility Criteria

Observational studies involving participants diagnosed with COPD that employed a control group/non-diseased group were included. Articles were deemed eligible when containing the following criteria: the evaluation of biomarkers in human blood samples, the presence of a control group with no statistical difference in age between diseased patients, or COPD patients who were age-matched with controls. The following were excluded from this SR: studies involving only animal populations, case reports, articles evaluating markers in patients with COPD exacerbation, studies involving respiratory infection, or those investigating immunological and pulmonary comorbidities associated with COPD.

### 2.4. Data Extraction

Data extraction was performed by two independent reviewers. All conflicts were resolved by a third reviewer. The following data were extracted: author, year of publication, study population, sample size, participant age, biomarker investigated, detection method, differences in marker findings between COPD patients and controls, correlations/associations with clinical parameters.

Full texts of selected articles were examined in detail, and appropriate data were extracted for analysis. Data were extracted in a standardized data extraction format prepared in Microsoft Excel. Reviewers contacted authors to obtain additional research data from all eligible studies included. 

### 2.5. Quality Analysis of Included Studies

The included observational studies were evaluated with respect to quality using the Newcastle–Ottawa Quality Assessment Scale [20]. This scale consists of three elements: selection, comparability, and Exposure/Outcome. As the maximum score on the Newcastle–Ottawa Quality Assessment Scale is nine points, studies scoring seven points or higher are considered to be of high quality. Each study was evaluated independently by the two reviewers using the Newcastle–Ottawa Quality Assessment Scale. Discrepancies were resolved by consensus of a third reviewer.

## 3. Results

### 3.1. Research and Study Selection

**Figure 1:**
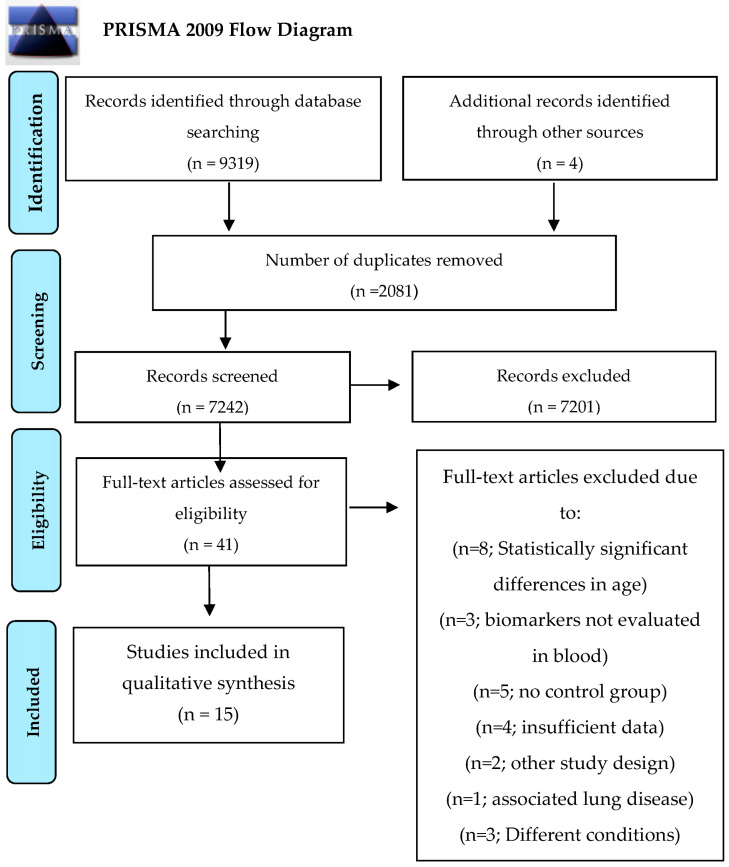
Flowchart of the included articles.

In all, 9323 records were identified during the search. After the removal of duplicate records, 7242 articles were maintained. Forty-one (41) were retained after the reading of titles and abstracts. Twenty-six (26) of these were excluded due to lacking eligibility criteria: eight studies were excluded as a result of statistically significant differences in age between COPD patients and controls [21,22,23,24,25,26,27,28,29]; three were excluded due to the investigation of biomarkers in lung or muscular tissue [30,31,32]; five studies had no control group [17,33,34,35,36]; three did not report the results of statistical testing for discrepancies in age between COPD patients and controls [37,38,39]; and one did not perform comparisons [40]. Two articles employed a study design that was inconsistent with the stated eligibility criteria [41,42]. One article was excluded due to the presence of an associated lung disease [43], and three others were not considered due to insufficient data according to the scope of the present SR [44,45,46]. Following the application of exclusion criteria, a total of 15 studies were included (Figure 1).

The characteristics of all included studies are shown in Table 1. Each study was characterized by two groups of interest (case and control), and findings on biomarkers were immediately extracted and uploaded to the database (Table 2). Ten articles demonstrated relationships between markers of immunosenescence and/or inflammation and clinical variables [47,48,49,50,51,52,53,54,55,56].

### 3.2. Study Quality

The risk of bias as evaluated by the Newcastle–Ottawa Quality Assessment Scale in cohort and case–control studies is shown in Supplementary Material. After a complete reading of the 15 selected articles, quality assessment revealed that eight articles scored between 7 and 9 points, indicating that 53% of the studies evaluated were of high quality. 

### 3.3. Inflammation and Immunosenescence in Chronic Obstructive Pulmonary Disease

In the context of senescence, four studies analyzed the levels of pro-inflammatory cytokines IL-6 and IL-8 in patients with COPD [54,56,57,58]. Three employed an ELISA, and one used flow cytometry to quantify cytokine production. One study measured cytokines in plasma following lipopolysaccharide stimulation in whole blood [57]. Three observational studies found significantly elevated IL-6 levels in the peripheral blood of a stable COPD group, suggesting greater systemic inflammatory activity compared to in controls without disease [54,56,58]. Moreover, one of these studies identified a negative correlation between IL-6 and TL [56]. Three studies investigating IL-8 identified statistical differences between COPD patients and controls [56,57,58]. In one of these, logistic regression analysis adjusted for age, sex, and number of pack-years demonstrated that IL-8 levels in the blood of COPD patients were not significantly different compared to in healthy subjects [56].

### 3.4. Naïve, Memory, and CD28^null^ T Cells

Six studies explored T cell subsets and markers of senescence [12]. Only one study reported on the distribution of naïve, central memory, and effector memory T cells. This study also evaluated cytomegalovirus (CMV) status in both COPD and control groups [12]. Fernandes et al. found higher percentages of CD4^+^ and CD8^+^ naïve T cells in COPD patients compared to age-matched groups of healthy subjects and smokers, reporting no significant differences in these subsets [12]. Regarding CD8^+^CD28^null^, four [48,49,50,59] studies showed a higher proportion of this subset in COPD patients compared to controls; one of the four [48] found no correlation between CD8^+^CD28^null^ and forced expiratory volume in 1 s (FEV_1_). Two out of five studies that investigated CD4^+^CD28^null^ T cell frequency reported no increases in this marker in patients with COPD compared to controls [48,50]. A study by Lambers et al. on COPD severity identified different frequencies of CD4^+^CD28^null^ T cells in the COPD III–IV groups compared to healthy individuals who smoked or not; however, no significant differences were seen between COPD I–II and the healthy individuals investigated [52].

### 3.5. Telomere Length in Circulating Leukocytes

TL in leukocyte subpopulations in peripheral blood was assessed by PCR. Four studies presented findings on relative telomere-to-single-copy-gene (T/S) ratio [47,51,53,54,55,56,58] (Table 1). All four studies found statistically significant differences in TL in subjects with COPD compared to controls. Conversely, Fernandes et al. reported shorter TL in CD4^+^ T cells from COPD patients, despite lacking statistical significance compared to smoker and healthy subjects, while differences were significant with respect to the CD8^+^ subset [60]. In studies evaluating TL, three out of four identified correlations with the repercussion of this marker on disease. TL was found to negatively correlate with age in COPD patients and positively with fat mass after adjusting for age and gender [51]. One study analyzing leukocyte TL over a 3-year follow-up period [47] reported telomere shortening in COPD patients, both at baseline and three years later, compared to age-matched smoker controls, which was not found to correlate with lung function [47].

## 4. Discussion

Senescent immune cells have been linked with pro-inflammatory cytokines in COPD patients when compared to controls [30,50,56,61], which may justify the maintenance of inflammation in this disease [30].

Decreased CD28 expression is considered a good marker of senescence status in the immune system [62]. In addition, inflammatory cytokines could be also considered markers of immunosenescence since a low-grade inflammatory state has been described during the aging process, even in the absence of infection by pathogens [63]. Many of these immunosenescence markers are present in COPD, as evidenced by most of the articles included in this SR.

In different age-related diseases, IL-6 and IL-8 are described as senescence-associated secretory phenotype markers and these cytokines were COPD-related also [64]. Most of the reviewed articles evaluated leukocyte TL, most likely due to its status as a traditional marker of immunosenescence. IL-6 and IL-8 were assessed in only four articles due to the increased production of these inflammatory cytokines during COPD. Although the inflammation reported in studies on COPD cannot be definitively attributed to only being a result of aging, our review exclusively considered studies with stable patients as one of the eligibility criteria. During episodes of disease exacerbation, higher levels of IL-6 [65] and IL-8 [66] are reported compared to in stable COPD, which precludes this state from being considered low-grade inflammation.

In three of the four studies evaluating IL-6 in COPD patients, increased levels were reported, which reinforces the role of this marker both in the context of this disease and in other age-related disorders [67]. Accordingly, the literature also found elevated IL-6 levels in stable COPD patients compared to controls [68,69]. However, Maté et al. did not identify statistical differences in IL-6 between COPD patients and controls, which could be explained by a low number of participants [57]. Another study by Zeng et al. investigated both IL-6 and IL-8 and concluded that results were not representative of the overall COPD population due to small sample size [70]. With regard to inflammation, IL-8 plays a crucial role as this cytokine is necessary for the recruitment and activation of neutrophils [71]. In COPD, senescent pulmonary vascular endothelial cells secrete pro-inflammatory cytokines, including IL-6 and IL-8 [30]. Alveolar senescent cells produce greater amounts of IL-6, IL-8, and TNF-α than pre-senescent cells, favoring chronic lung inflammation [72]. Thus, the low-grade inflammatory state observed during aging may potentially exacerbate the already existing inflammation in COPD, thus contributing to the worsening of lung function as well as comorbidities.

Two out of four studies comparing the distribution of CD4^+^CD28^null^ T cells found significantly higher frequencies in COPD patients compared to controls [52,59]. By contrast, all four articles evaluating CD8^+^CD28^null^ found higher proportions in COPD patients [48,49,50,59].

During aging, CD8^+^ T cells exhibit a greater rate of phenotypic change, characterized by a more frequent loss of CD28 expression, than CD4^+^ T cells. This loss of CD28 may correspond to a median frequency of around 7% in CD4^+^CD28^null^ T cells, and approximately 53% in CD8^+^CD28^null^ T cells in individuals aged 60 years or older [73]. In the study subjects considered by this SR, more than 50% of COPD patients were over 60. One study did not mention patient age range despite utilizing age-matching methodology [59]. Interestingly, although a meta-analysis of the data was not performed, it is notable that three studies by Hodge et al. reported relative CD8^+^CD28^null^ expression above or equal to 53% in COPD patients [48,49,50].

Only one study meeting the eligibility criteria of this SR evaluated memory T cells, identifying an increased proportion of CD8^+^ central memory cells in individuals with COPD [12]. Throughout life, levels of circulating memory T cells typically remain stable, with increases noted around 60–65 years of age [74]. Latent persistent human CMV infection has been associated with immunosenescence through the reduction in naïve CD8^+^T cell frequencies and accumulation of memory T cells [75]. Only two [12,59] of the included studies evaluated seropositivity for CMV. Tan et al. propose that CMV reactivation is related to an increase in the number of CD28^null^ T cells in COPD, which results in enhanced systemic inflammation [59]. Phenotypic alterations in naïve and memory T cell subsets are another change that can impact adaptative immunity in COPD patients.

The literature contains an abundance of investigations on leukocyte TL. The fact that all articles in this SR studied the shortening of leukocyte telomeres in COPD suggests a possible pattern of accelerated immunosenescence associated with this disease. This notion is further supported by a meta-analysis of nine European studies conducted by Albrecht et al. [76]. Interestingly, none of the studies analyzed by Albrecht et al. was included in the present SR. Another meta-analysis, conducted by Wang et al. in 2022, reaffirmed that telomere shortening may indeed be a biomarker of accelerated aging in COPD [77].

Investigating the possible effects of immunosenescence markers related to disease severity, [26] reported a significant negative correlation between FEV_1_ and the percentage of glucocorticoid receptor-negative CD8^+^ CD28^null^ T cells in COPD patients [26]. On the other hand, Rode et al. (2013) speculated that accelerated cell turnover in response the inflammation caused by exposure to noxious particles or gases may lead to a positive association between telomere length and lung function [34].

Our study suffers from limitations related to the methodological aspects of some of the studies such as small sample size and heterogeneous subgroups, and approximately 47% of the studies did not report central tendency measures in the results (i.e., mean or median, SD, etc.) for some groups or in all samples. Despite our attempts to contact the study authors, we were unable to access the raw data to conduct further analysis. This, consequently, made it impossible to perform a meta-analysis. Unfortunately, it was not possible to definitively determine the magnitude of these changes through rigorous statistical analysis.

## 5. Conclusions

Considering the data available for this SR, our results indicate that changes associated with immunosenescence are observed in stable COPD patients, mainly increased IL-6 production and CD28^null^ T cell frequencies and TL shortening in leukocytes. Although lung function severity was the disease marker most frequently analyzed in the studies, the authors reached different conclusions with regard to the importance of this clinical marker and immunosenescence. Further study on other markers of disease is necessary to more comprehensively evaluate the implications of these changes in COPD.

## Figures and Tables

**Table 1:** Characteristics of the studies included in the systematic review.

Reference	Marker(s)	Method(s)	N Total	COPD		Control	
				n	Age, Years	n	Age, Years
Rutten et al., 2016 [54]	IL-6, IL-8, and Leukocyte TL	PCR and ELISA	280	160	62 ± 7	82	S: 62 ± 6
						38	NS: 59 ± 8
Savale et al., 2009 [56]	IL-6, IL-8, and Leukocyte TL	PCR and ELISA	291	136	62.9 ± 6.6	133	S: 62.2 ± 7.7
						42	NonS: 61.4 ± 6.1
Maté el al., 2021 [57]	IL-6 and IL-8	ELISA	37	22			
			Moderate: 63.57 ± 6.08	15	63.35 ± 5.02		
Boyer et al., 2015 [58]	IL-6, IL-8, and Leukocyte TL	PCR and Flow Cytometry	301	100	60.6 (56.7–65.9)	100	S: 59.6 (53.6–64.1)
						101	NonS: 59.5 (53.3–63.6)
Hodge et al., 2011 [48]	CD8^+^ and CD4^+^CD28^null^	Flow Cytometry	97	30	Curr-smokers: 59 ± 7	34	C: 53 ± 12
				18	Ex-smokers: 60 ± 6	15	S: 54 ± 12
Hodge et al., 2020 [49]	CD8^+^CD28^null^	Flow Cytometry	20	10	58 (±16)	10	56 (±9)
Hodge et al., 2022 [50]	CD4^+^ and CD8^+^CD28^null^	Flow Cytometry	21	10	54 (39–69)	11	56 (44–68)
Lambers et al., 2009 [52]	CD4^+^CD28^null^	Flow Cytometry	64	19	GOLD I-II: 60.68 ± 7.39	15	H: 57.20 ± 12.50
				16	GOLD III-IV: 58.31 ± 8.75	14	HS: 56.64 ± 9.17
Tan et al., 2016 [59]	CD8^+^ and CD4^+^CD28^null^	Flow Cytometry	66	33	Age-matched	33	Age-matched
Fernandes et al., 2022 [12]	Naïve and memory cells	Flow Cytometry	72	21	65 ± 5.1	29	H: 65.4 ± 2.9
						22	S: 64.2 ± 3.3
Córdoba-Lanús et al., 2017 [47]	Leukocyte TL	PCR	242	121	57 ± 8	121	57 ± 8
Houben et al., 2009 [51]	Leukocyte TL	PCR	122	102	62.9 ± 9.3	20	60.7 ± 3.5
Moon et al., 2021 [53]	Leukocyte TL	PCR	446	285	72.86 ± 7.01	161	71.96 ± 7.22
Sadr et al., 2015 [55]	Leukocyte TL	PCR	169	84	64.33 ± 10.04	85	65.06 ± 10.04
Fernandes et al., 2021 [60]	Leukocyte TL	PCR	75	24	Age-matched	51	Age-matched

Abbreviations: S = smokers; NS = never smoked; NonS = non-smokers; H = healthy subjects; HS = healthy smoker; Curr-smokers = current smokers; Ex-smokers = former smokers; N/R = not reported; TL = telomere length. Data expressed as medians (interquartile range) or mean ± SD for continuous variables or absolute values for categorical variables.

**Table 2:** Characteristics of studies investigating biomarkers in COPD.

Reference	Marker	COPD	Control	*p*	Implication(s) for Disease
Savale et al., 2009 [56]	IL-6	2.4 (0.3–30.5)	S:1.5 (0.5–15.3)	0.0001	Correlated negatively with TL
			NonS: 0.9 (0.1–3.6)		
				Adj. Patients vs. Control: 0.003	
Boyer et al., 2015 [58]	IL-6	16.5 (14.3–19.2)	S: 15.7 (12.9–18.5)	0.01	N/R
			NonS: 14.7 (12.9–17.3)		
				COPD vs. Smokers: 0.15	
Ruttern et al., 2016 [54]	IL-6	4.8 (2.7–8.6)	S: 2.4 (1.6–4.9)	<0.01	N/R
			NS: 2.7 (1.7–5.0)	<0.01	
Maté et al., 2021 [57]	IL-6	Severe: ↑	↓	Not significant	N/R
Savale et al., 2009 [56]	IL-8	12.4 (0.9–36.6)	S: 9.6 (3.6–37.6)	0.0001	N/R
			NonS: 8.3 (2.2–19.2)		
				Adj. Patients vs. Control: 0.069	
Boyer et al., 2015 [58]	IL-8	48.8 (42.7–53.4)	S: 47.0 (40.0–51.6)	0.008	N/R
			NonS: 43.5 (38.9–50.4)		
				COPD vs. S: 0.11	
Rutten et al., 2016 [54]	IL-8	9.9 (6.1–14.9)	S: 6.8 (4.6–13.1)	Not significant	N/R
			NS: 6.7 (3.7–9.6)	Not significant	
Maté et al., 2021 [57]	IL-8	Moderate: ↑	↓	0.031	N/R
		Severe: ↑		0.012	
Tan et al., 2016 [59]	CD4^+^CD28^null^	↑	↓	0.02	N/R
	CD8^+^CD28^null^	↑	↓	0.005	
Lambers et al., 2009 [52]	CD4^+^CD28^null^	GOLD I-II: 3.22 (1.83–4.62)	H: 1.96 (1.07–2.84)	GOLD I–II vs. HS: 0.046	Negative correlation between CD4^+^CD28^null^ and FEV_1_ and MEF50%; CD4^+^CD28^null^ cells exhibited high predictive power for COPD diagnosis
		GOLD III-IV: 7.53 (2.67–12.39)	HS: 1.5 (0.41–2.59)	GOLD III-IV vs. H: 0.012	
				GOLD III-IV vs. HS: 0.002	
Fernandes et al., 2022 [12]	EM TCD8^+^	↓	S: ↑	<0.01	N/R
	CM TCD8^+^	↓	S: ↑	<0.01	
	N.CD4^+^CD27^-^CD28^null^	↑	S: ↓	<0.001	
	CM CD4^+^CD27^-^CD28^null^	↑	H: ↓	<0.05	
Hodge et al., 2022 [50]	CD8^+^CD28^null^	55 (38–63)	34 (18–42)	<0.05	Negative correlation between CD8^+^CD28^null^ expressing GCR and FEV_1_
	CD4^+^CD28^null^	8 (3–12)	6 (3–12)	Not significant	
Hodge et al., 2011 [48]	CD8^+^CD28^null^	Curr-smokers: ↑	↓	<0.05	No correlation between CD8^+^CD28^null^ and FEV_1_
		Ex-smokers: ↑			
	CD4^+^CD28^null^	Curr-smokers: ↑	↓	Not significant	
		Ex-smokers: ↑			
Hodge et al., 2020 [49]	CD8^+^CD28^null^	57 ± 8.4	33 ± 8.5	Statistically significant	Positive correlation between SIRT1 CD8^+^CD28^null^ T cells and FEV_1_
Houben et al., 2009 [51]	Leukocyte TL	↓	↑	<0.05	Negative correlation between TL and age; positive correlation with BMI
Savale et al., 2009 [56]	Leukocyte TL	0.57 (0.23–1.18)	S: 0.79 (0.34–1.58)	0.0001	Positive correlation between TL and PaO_2_, SaO_2_, and 6MWT; negative correlation with age, PaCO_2_, and IL-6
			NonS: 0.85 (0.38–1.55)		
Boyer et al., 2015 [58]	Leukocyte TL	0.37 (0.31–0.4)	S: 0.43 (0.36–0.50)	0.000	N/R
			NonS: 0.42 (0.36–0.51)		
Sadr et al., 2015 [55]	Leukocyte TL	0.61 ± 0.08	0.69 ± 0.09	<0.001	Correlation between BMI and TL not significant
Rutten et al., 2016 [54]	Leukocyte TL	4.4 (4.0–4.7)	S: 4.6 (4.1–5.2)	<0.01	Association between TL and FEV_1_
			NS: 4.7 (4.2–5.1)	<0.01	
Córdoba- Lanús et al., 2017 [47]	Leukocyte TL	0.68 ± 0.25	0.88 ± 0.52	<0.0001	No significant relationships between the rate of changein TL and lung function
				Adj. Patients vs. Control: *p* = 0.003	
Fernandes et al., 2021 [12]	Leukocyte TL	↓	S:↑	<0.05	N/R
Moon et al., 2021 [53]	Leukocyte TL	16.81 ± 13.90	21.97 ± 14.43	<0.001	No significant association between TL and COPD exacerbation

Abbreviations: ↑ higher; ↓ lower; S = smokers; NS = never smoked; NonS = non-smokers; H = healthy subjects; HS = healthy smoker; N/R = not reported; TL= telomere length; Adj = adjusted statistical analysis; 6MWT = 6 min walk test. Data expressed as medians (interquartile range) or mean ± SD for continuous variables or absolute values for categorical variables. Data are expressed as medians (interquartile ranges) or mean ± SD for continuous variables and numbers (percentages) for categorical variables.

## Data Availability

Relevant data are contained within the article. Additional data are available from the corresponding author upon reasonable request.

## Supplementary Materials

The following are available online at https://www.mdpi.com/article/10.3390/jcm13123449/s1, Table S1: Quality assessment and risk of bias for included studies applying the Newcastle-Ottawa Scale.
