# Supplementary material for: Immunosenescence and Inflammation in Chronic Obstructive Pulmonary Disease: A Systematic Review
Source: J Clin Med. 2024 Jun 13;13(12):3449. doi: 10.3390/jcm13123449 (PMC11205253; doi:10.3390/jcm13123449)
Supplement: Supplementary file 1 [file jcm-13-03449-s001.zip › jcm-2998604-supplementary.pdf]

Table S1: Quality assessment and risk of bias for included studies applying the Newcastle-Ottawa Scale.

Reference	Design	Selection	Comparability	Exposure	Total
Boyer et al., 2015.[58]	CASE-CONTROL	4	1	2	7
Córdoba-Lanús et al., 2017.[47]	COHORT	4	2	3	9
Fernandes et al., 2022.[12]	CASE-CONTROL	2	2	2	6
Fernandes et al. ,2021.[60]	CASE-CONTROL	3	2	3	8
Hodge, et al., 2011.[48]	CASE-CONTROL	3	1	2	6
Hodge et al., 2020.[49]	CASE-CONTROL	3	2	2	7
Hodge et al., 2022.[50]	CASE-CONTROL	2	2	2	6
Houben et al., 2009.[51]	CASE-CONTROL	3	1	2	6
Lambers et al., 2009.[52]	CASE-CONTROL	2	2	2	6
Maté et al., 2021.[57]	CASE-CONTROL	1	2	2	5
Moon et al., 2021.[53]	CASE-CONTROL	4	1	3	8
Rutten et al., 2016.[54]	CASE-CONTROL	4	2	2	8
Sadr et al., 2015.[55]	CASE-CONTROL	3	2	3	8
Tan et al., 2016.[59]	CASE-CONTROL	4	2	1	7
Savale et al., 2009.[56]	CASE-CONTROL	2	2	2	6
